# Superior Trunk Block Is an Effective Phrenic-Sparing Alternative to Interscalene Block for Shoulder Arthroscopy: A Systematic Review and Meta-Analysis

**DOI:** 10.7759/cureus.48217

**Published:** 2023-11-03

**Authors:** Sara Amaral, Rafael Arsky Lombardi, Heitor Medeiros, Alleh Nogueira, Jeff Gadsden

**Affiliations:** 1 Anesthesiology, Hospital Regional Deputado Afonso Guizzo, Ararangua, BRA; 2 Anesthesiology, University of Nebraska Medical Center, Omaha, USA; 3 Anesthesiology, Hospital Universitário Onofre Lopes, Natal, BRA; 4 Anesthesiology, Escola Bahiana de Medicina e Saúde Pública, Salvador, BRA; 5 Anesthesiology, Duke University Medical Center, Durham, USA

**Keywords:** superior trunk block, interscalene block, shoulder arthroscopy, brachial plexus block, regional anesthesia

## Abstract

The interscalene block (ISB) is the standard regional anesthesia for shoulder arthroscopy. However, the superior trunk block (STB) is an alternative with a potentially safer profile. This meta-analysis aimed to compare the incidence and degree of hemidiaphragmatic paralysis and block efficacy of these techniques. We searched MEDLINE, EMBASE, Scopus, and Cochrane databases to identify randomized controlled trials (RCTs). The main outcome was total hemidiaphragmatic paralysis. We used the Grading of Recommendation, Assessment, Development, and Evaluation (GRADE) framework to assess the certainty of evidence.

Four RCTs and 359 patients were included. The STB group showed lower total hemidiaphragmatic paralysis (RR 0.07; 95% CI 0.04 to 0.14; *p*<0.0001). The incidence of subjective dyspnea (*p* = 0.002) and Horner's syndrome (*p*<0.001) was significantly lower with STB relative to ISB. There was no significant difference between groups in block duration (p = 0.67). There was a high certainty of evidence in the main outcome as per the GRADE framework. Our findings suggest that STB has a better safety profile than ISB, resulting in lower rates of hemidiaphragmatic paralysis and dyspnea while providing a similar block. Therefore, STB could be preferred to ISB, especially in patients susceptible to phrenic nerve paralysis complications.

## Introduction and background

Shoulder arthroscopy is a frequently performed procedure that leads to substantial postoperative pain. Effective pain management is crucial for ensuring patient comfort and successful rehabilitation. Regional anesthesia techniques, such as the interscalene block (ISB), have been commonly used to manage postoperative pain. However, ISB has been associated with adverse effects such as phrenic nerve paralysis and subsequent respiratory depression, particularly in patients with pre-existing respiratory disease. Therefore, comprehending the risk factors and incidence of these complications is essential for selecting the appropriate anesthesia technique and reducing patient morbidity [1,2].

In 2014, Burckett-St. Laurent et al. proposed using superior trunk block (STB) as a substitute for ISB to avoid phrenic nerve involvement. By precisely targeting the superior trunk of the brachial plexus at a more inferolateral location, STB has the potential to provide equivalent pain relief while reducing the likelihood that local anesthetic will spread to the phrenic nerve. Avoiding phrenic nerve involvement in STB can decrease the risk of respiratory depression and enhance patient safety, particularly in those with underlying lung disease or compromised respiratory function [3,4].

The efficacy and safety of STB and ISB have been evaluated in several studies for postoperative pain management after shoulder arthroscopy. However, the results of these studies are inconsistent, and the optimal regional anesthesia technique for this procedure remains to be determined [5-7].

It is unknown whether the anatomical advantage of STB corresponds to improved outcomes. We then conducted a systematic review and meta-analysis of RCTs to compare the STB versus the ISB in patients undergoing shoulder arthroscopy. The main goal was to assess the incidence and extent of hemidiaphragmatic paralysis and its associated consequences. Additionally, we aimed to evaluate the efficacy of the blocks and determine whether the STB could offer a safer alternative while maintaining comparable effectiveness to the ISB.

This article was previously presented as a meeting abstract at the 6th World Congress of Regional Anesthesia and Pain Medicine on September 7, 2023.

## Review

Methods

We registered this meta-analysis prospectively under protocol number CRD42023406892 in the International Prospective Register of Systematic Reviews (PROSPERO). Data registration, conduction of the meta-analysis, and reporting were compliant with the Cochrane Handbook for Systematic Reviews of Interventions [8] and the Preferred Reporting Items for Systematic Reviews and Meta-Analyses (PRISMA) guidelines [9].

Eligibility Criteria

The following eligibility criteria were used for this meta-analysis: (1) RCTs; (2) comparison of ultrasound-guided ISB with STB; (3) patients older than 18 years undergoing arthroscopic shoulder surgery; and (4) reporting any of the clinical outcomes of interest. We excluded: (1) overlapping populations, defined as studies with overlapping institutions and recruitment periods; (2) non-randomized studies; (3) conference abstracts; and (4) non-US-guided diaphragm paralysis evaluations. There were no restrictions based on the date or language of publication. In cases of missing data from individual studies, the corresponding authors were contacted for specific study results.

Search Strategy and Data Extraction

MEDLINE, EMBASE, Scopus, and the Cochrane Central Register of Controlled Trials were systematically searched for RCTs meeting the eligibility criteria published from inception to March 2023. The search strategy consisted of ("upper trunk" OR "superior trunk") AND interscalene AND (block OR blockade). It was independently conducted by two different authors (SA and RL). Disagreements were resolved by consensus. We last searched all databases on April 16, 2023. In addition, references from all included studies were reviewed in the search for additional studies. The gray literature was not searched. Three authors (SA, RL, and HM) independently extracted data. Author disagreements were resolved through consensus.

Outcomes

The outcomes of interest were (1) incidence of total and absent hemidiaphragmatic paralysis; (2) percentile reduction of diaphragmatic excursion at 30 minutes; (3) reduction of diaphragmatic excursion at 30 minutes in cm; (4) duration of motor block; (5) duration of sensory block; (6) cumulative opioid consumption in 24 hours; (7) pain score at rest at 24 hours; (8) patient satisfaction with analgesia at 24 hours; (9) incidence of subjective dyspnea; and (10) incidence of Horner syndrome. In all of the included studies, the diaphragmatic function was evaluated using ultrasound M-Mode, and complete hemidiaphragmatic paralysis was defined as a >75% decrease from baseline in diaphragmatic movement or the occurrence of paradoxical movement. Cumulative opioid consumption was reported in oral morphine equivalents. In each study, pain scores were measured using the numeric rating scale (NRS) or the visual analog scale (VAS). Patient satisfaction with analgesia was assessed using a numerical rating scale from one to ten. Whenever at least three RCTs had results for an endpoint, a meta-analysis with pooled results was performed.

Quality Assessment

As only RCTs were included, we used version two of the Cochrane Risk of Bias assessment tool (RoB 2) to evaluate the risk of bias [10]. The risk assessment was performed by two independent authors (SA and HM), and disagreements were resolved through consensus between them. Publication bias could not be assessed with funnel plots or the Egger test because only four studies were included. Inspection of funnel plots has limited value when the sample size is small, and the Egger test is not recommended until at least 10 studies are summarized [11]. Nevertheless, we included a Doi plot and LFK index [12,13]. P-values < 0.05 were considered statistically significant. The results were subjected to sensitivity analysis of the source of heterogeneity by systematically removing each study from the pooled analysis, one at a time, and recalculating the mean difference (MD) between groups. Two independent authors assessed the quality of the evidence with the GRADEpro software (Evidence Prime) [14]. The GRADE (Grading of Recommendations, Assessment, Development, and Evaluations) criteria were employed during the evaluation process [15].

Data Analysis

Treatment effects for continuous outcomes were compared using MD with 95% confidence intervals (CI). Treatment effects for categorical outcomes were compared using risk ration (RR) with 95% confidence intervals (CI). For pain scores, we used 11mm as the minimally clinically important difference (MCID) [16]. We used Review Manager 5.4 (Nordic Cochrane Center, The Cochrane Collaboration, Copenhagen, Denmark) to perform the statistical analysis. We assessed heterogeneity using the Cochran Q test and I2 statistics, and we also used a random-effect model.

Results

Study Selection and Characteristics

**Figure 1:**
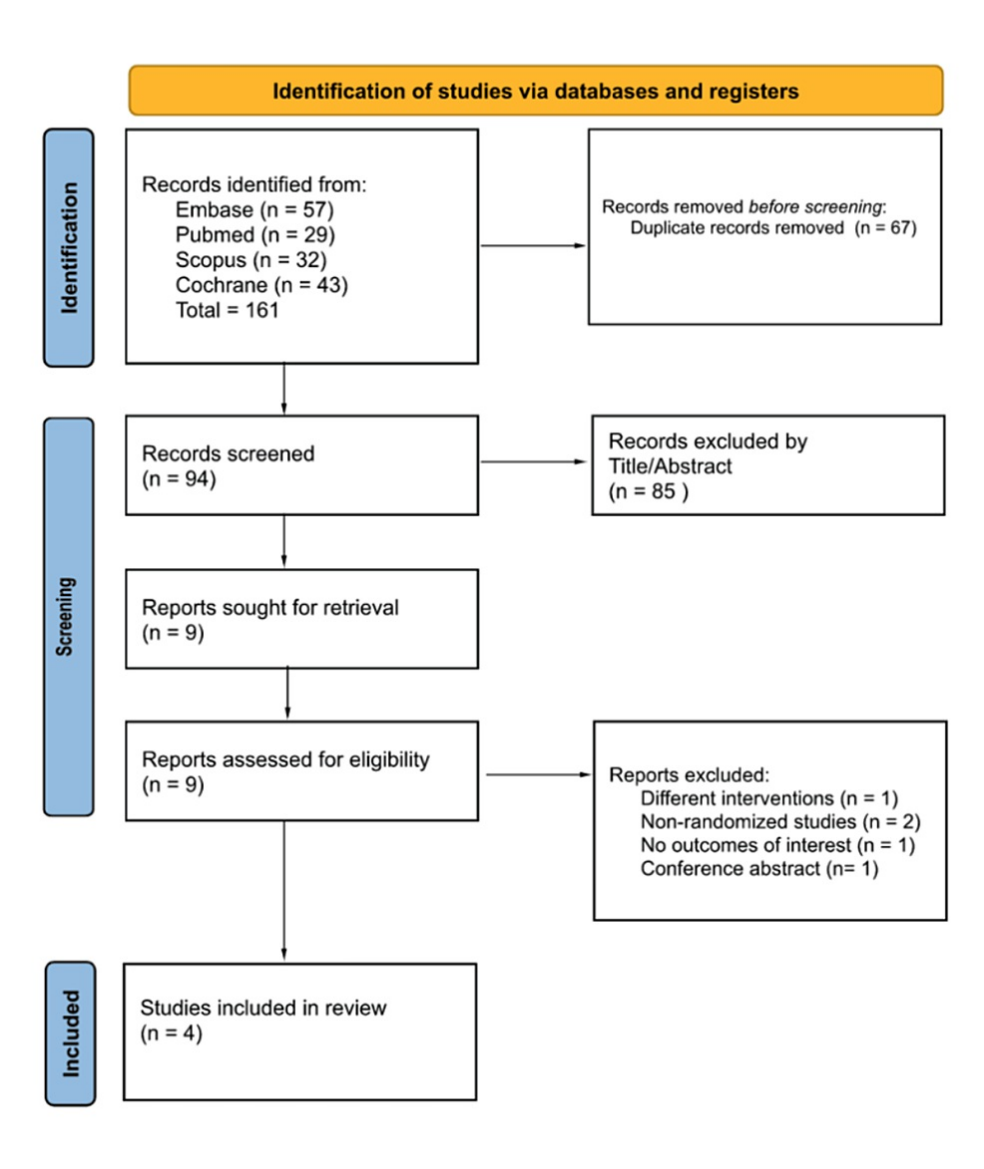
PRISMA flow diagram of study screening and selection

In total, 161 articles were retrieved in the search, of which 67 were duplicates and 85 were deemed unrelated based on their titles or abstracts. A thorough screening was conducted on the remaining nine articles, and only four were included in the meta-analysis after considering the inclusion and exclusion criteria (Figure 1).

Table 1 presents the baseline characteristics of individual studies. This review included 359 adult patients, with 56.5% being male. A total of 178 (49.5%) patients were randomized to receive the STB.

**Table 1:** Baseline characteristics of included studies ISB: interscalene block; SD: standard deviation; RCT: randomized controlled trial; STB: superior trunk block

Study	Design	Patients,n STB/ISB	Female,n STB/ISB	Age in years (mean±SD)		Local anesthetic	Volume of local anesthetic in mL STB/ISB	Concentration of local anesthetic	Types of Arthroscopic Procedures n STB/ISB	Follow-up
				STB	ISB					
Kang et al., 2019 [17]	RCT	38/40	15/20	55±14	53±14.8	Ropivacaine	15mL/15mL	0.5%	Rotator cuff repair (30/28); Bankart repair (3/5); Superior labrum repair (3/3); Latarjet (2/4)	14 days
Kim et al., 2019 [18]	RCT	62/63	14/19	49.4±16.3	48.9±15	Bupivacaine	15mL/15mL	0.5%	Rotator cuff repair (30/31); Non-Rotator cuff repair (32/32)	7 days
Yin et al., 2020 [19]	RCT	30/30	13/15	52±20	47±20	Ropivacaine	15mL/15mL	0.375%	Rotator cuff repair (18/20); Other (12/10)	1 day
Zhang et al., 2022 [20]	RCT	48/48	33/28	59.8±10.4	60.4±9.1	Ropivacaine	5mL/15mL	0.5%	Rotator cuff repair (36/34); Acromioplasty (43/45); Biceps tenotomy (7/5); SLAP repair (0/3); Bankart repair (1/0)	7 days

Hemidiaphragmatic Paralysis

**Figure 2:**
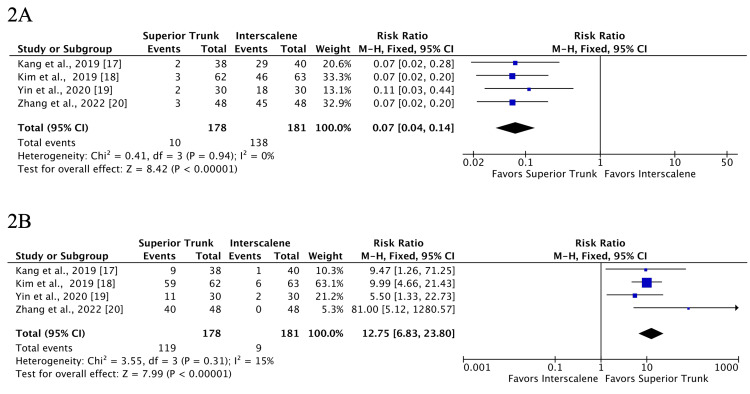
The incidence of total hemidiaphragmatic paralysis 30 minutes after block (A) and the absence of hemidiaphragmatic paralysis (B) both favored the superior trunk block when compared to the interscalene block

The incidence of total hemidiaphragmatic paralysis 30 minutes after the block was significantly lower for STB compared with ISB (RR 0.07; 95% CI 0.04 to 0.14; p < 0.0001; I2 = 0%; 4 RCTs, 359 patients) (Figure 2a). Moreover, the absence of hemidiaphragmatic paralysis was significantly higher for STB (RR 12.75; 95% CI 6.83 to 23.80; p<0.00001; I2=15%; 4 RCTs, 359 patients) (Figure 2b).

Additionally, diaphragmatic excursion at 30 minutes was significantly higher for STB (MD 2.77cm; 95% CI 1.6 to 3.94cm; p<0.00001; I2 = 96%; 4 RCTs, 359 patients). The reduction in diaphragmatic excursion at 30 minutes compared with baseline significantly favored the STB group (MD -0.44; 95% CI -0.64 to -0.23; p<0.0001; I2 = 97%; 4 RCTs, 359 patients).

Duration of Block, Pain Score and Cumulative Opioid Consumption in 24 Hours

**Figure 3:**
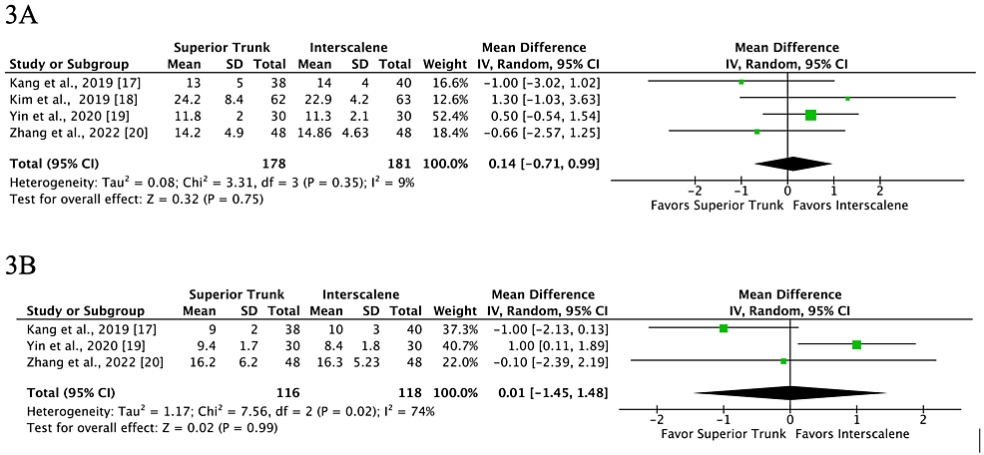
Motor block (A) and analgesia (B) durations in hours showed no significant difference between superior trunk block and interscalene block

Our study did not find any statistically significant difference between the ISB and STB in terms of motor block duration (MD 0.17 hours; 95% CI -0.61 to 0.95 hours; p = 0.67; I2 = 9%; 4 RCTs, 359 patients) (Figure 3A); duration of analgesia (MD 0.01 hours; 95% CI -1.45 to 1.48 hours; p = 0.99; I2 = 74%; 3 RCTs, 234 patients) (Figure 3B); and cumulative opioid consumption in 24 hours (MD -0.14mg; 95% CI -0.45 to 0.18mg; p = 0.4; I2 = 47%; 3 RCTs, 299 patients). However, we found that pain scores at rest at 24 hours were significantly lower with STB (MD -0.75 mm; 95% CI -1.35 to -0.15 mm; p = 0.01; I2 = 44%; 3 RCTs, 299 patients), although it did not reach the minimum clinically important difference of 11 mm.

Subjective Dyspnea and Horner Syndrome

Subjective dyspnea was significantly decreased in the STB group (RR 0.24; 95% CI 0.10 to 0.60; p = 0.002; I2 = 0%; 4 RCTs; 359 patients), as well as the incidence of Horner syndrome (RR 0.06; 95% CI 0.01 to 0.32; p<0.001; I2 = 0%; 3 RCTs; 221 patients).

Patient Satisfaction

There was no significant difference between groups in patient satisfaction with analgesia at 24 hours (MD -0.04; 95% CI -0.26 to 0.18; p = 0.72; I2 = 0%; 3 RCTs; 299 patients).

Quality assessment 

**Figure 4:**
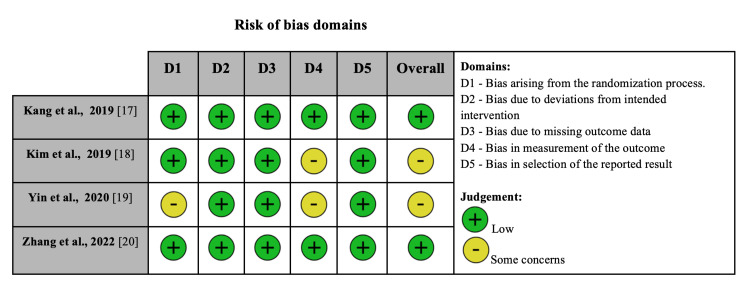
Risk of bias summary for randomized studies (RoB 2)

Figure 4 summarizes the individual evaluation of each RCT included in the meta-analysis using the RoB 2 quality assessment tool [10].

Two included studies were rated as having a low risk of bias and two as having some concerns. Sensitivity analysis with the removal of each individual study did not change the overall conclusion in any of the outcomes evaluated. The GRADE assessment is displayed in Tables 2, 3.

**Table 2:** Summary of findings RCT: randomized controlled trial High certainty: We are very confident that the true effect lies close to that of the estimate of the effect. Moderate certainty: We are moderately confident in the effect estimate. The true effect is likely to be close to the estimate of the effect, but there is a possibility that it is substantially different. Low certainty: Our confidence in the effect estimate is limited. The true effect may be substantially different from the estimate of the effect. Very low certainty: We have very little confidence in the effect estimate. The true effect is likely to be substantially different from the estimate of the effect.

Outcome	No of studies	Study design	Risk of bias	Inconsistency	Indirectness	Imprecision	Certainty
Total hemidiaphragmatic paresis	4	RCT	Not serious	Not serious	Not serious	Not serious	⨁⨁⨁⨁ High
No hemidiaphragmatic paresis	4	RCT	Not serious	Not serious	Serious	Serious	⨁⨁⨁◯ Moderate
Diaphragmatic excursion at 30min	4	RCT	Not serious	Very serious	Serious	Serious	⨁◯◯◯ Very low
Diaphragmatic excursion reduction at 30min	4	RCT	Not serious	Very serious	Not serious	Not serious	⨁⨁◯◯ Low
Subjective dyspnea	4	RCT	Not serious	Not serious	Not serious	Serious	⨁⨁⨁⨁ High
Horner syndrome	3	RCT	Not serious	Not serious	Not serious	Serious	⨁⨁⨁⨁ High
Pain score at 24h	3	RCT	Not serious	Serious	Not serious	Not serious	⨁⨁⨁◯ Moderate
Opioid consumption at 24h	3	RCT	Not serious	Serious	Not serious	Not serious	⨁⨁⨁◯ Moderate
Duration of analgesia	3	RCT	Not serious	Not serious	Serious	Not serious	⨁◯◯◯ Very low
Duration of motor block	4	RCT	Not serious	Not serious	Serious	Serious	⨁⨁⨁◯ Moderate
Patient satisfaction	3	RCT	Not serious	Not serious	Not serious	Not serious	⨁⨁⨁⨁ High

**Table 3:** Evidence profile: superior trunk block vs interscalene block for shoulder arthroscopy STB: superior trunk block; ISB: interscalene block; SD: standard deviation; GRADE: Grading of Recommendation, Assessment, Development, and Evaluation; RCT: randomized controlled trial

Population: Adult patients undergoing shoulder arthroscopy intervention: Superior trunk block comparator- Interscalene block						
Outcomes	STB Mean (SD)	ISB Mean (SD)	Mean difference or risk ratio (95% Confidence Interval)	Number of participants (studies)	Quality or certainty of the evidence (GRADE)	Comments
Total hemidiaphragmatic paresis	10/178	138/181	0.07 (0.04-0.14)	359 (4 RCTs)	⨁⨁⨁⨁ High	Low heterogeneity
No hemidiaphragmatic paresis	119/178	9/181	12.75 (6.83-23.80)	359 (4 RCTs)	⨁⨁⨁◯ Moderate	Low heterogeneity
Diaphragmatic excursion at 30min	3.57 (1.50)	0.80 (1.19)	2.77 (1.60-3.94)	359 (4 RCTs)	⨁◯◯◯ Very low	I^2^ Test for heterogeneity 96%
Diaphragmatic excursion reduction at 30min	0.41 (0.37)	0.87 (0.22)	-0.44 (-0.64- -0.23)	359 (4 RCTs)	⨁⨁◯◯ Low	I^2^ Test for heterogeneity 97%
Subjective dyspnea	5/178	23/181	0.24 (0.10-0.60)	359 (4 RCTs)	⨁⨁⨁⨁ High	Low heterogeneity
Horner syndrome	1/140	36/141	0.06 (0.01-0.24)	281 (3 RCTs)	⨁⨁⨁⨁ High	Low heterogeneity
Pain score at 24h	3.36 (2.22)	4.18 (2.44)	-0.75 (-1.35- -0.15)	299 (3 RCTs)	⨁⨁⨁◯ Moderate	I^2^ Test for heterogeneity 44%
Opioid consumption at 24h	23.25 (24.87)	25.69 (24.25)	-0.14 (-0.45- 0.18)	299 (3 RCTs)	⨁⨁⨁◯ Moderate	I^2^ Test for heterogeneity 47%
Duration of analgesia	12.08 (5.37)	12.19 (5.21)	0.01 (-1.47-1.49)	234 (3 RCTs)	⨁◯◯◯ Very low	I^2^ Test for heterogeneity 74%
Duration of motor block	17.02 (8.05)	16.88 (6.06)	0.17 (-0.61-0.95)	359 (4 RCTs)	⨁⨁⨁◯ Moderate	Low heterogeneity
Patient satisfaction	8.44 (2.15)	8.48 (2.17)	-0.04 (-0.26-0.18)	299 (3 RCTs)	⨁⨁⨁⨁ High	Low heterogeneity

Publication bias analyzed by the Doi plot showed asymmetry and Luis Furuya-Kanamori (LFK) index was 2.23.

Discussion

This is the first meta-analysis to pool the results of RCTs comparing STB with ISB in patients undergoing shoulder arthroscopy. The study included four RCTs and 359 patients. Overall, the STB proved equally effective as the ISB regarding analgesia while demonstrating a higher safety profile. Total and absent hemidiaphragmatic paralysis endpoints suggested that STB provides less interference with respiratory function when compared to the ISB. These findings were further supported by the consistent absence of statistical evidence for differences between the two blocks in most analgesic outcomes, such as opioid consumption, analgesia time, and overall patient satisfaction with analgesia. The STB was also superior to the ISB in pain scores at rest at 24 hours.

The ISB has traditionally been used as the preferred technique for shoulder surgery due to its proven efficacy in anesthesia and pain management. However, ISB is associated with several complications, the most prominent of which is hemidiaphragmatic paralysis [21,22]. Studies have hypothesized that a low-volume ISB [23,24], a two-point injection ISB [25], or a lower ISB may reduce the incidence of phrenic nerve paralysis [26]. However, none of these scenarios have eliminated this risk. This is because the phrenic nerve lies within 2 mm of the brachial plexus at the level of the cricoid cartilage. As the phrenic nerve moves caudally, it diverges from the plexus. Therefore, it is plausible that a more distal administration of the local anesthetic would further decrease the risk of phrenic nerve paralysis [27,28].

Recent studies suggest that the STB is a viable alternative for patients who cannot undergo ISB due to the risk of respiratory complications [3,26,29-34]. A cadaveric dye spread study using 5 mL of dye demonstrated staining of the superior trunk in all cases but not of the phrenic nerve [35]. However, another cadaveric study using 25 mL of dye reached the phrenic nerve in 57% of all cases [36]. These findings suggest that it is essential to consider the distance from the phrenic nerve and a relatively small volume of local anesthetic to minimize phrenic nerve paralysis.

Our findings are consistent with other contemporary trials of ISB for shoulder arthroscopy. A prior RCT including 72 patients who underwent ISB showed an 88.9% incidence of phrenic nerve paralysis, similar to the 95% incidence shown in our meta-analysis [30]. Furthermore, several meta-analyses comparing the ISB with other upper limb blocks for shoulder surgery have reported similar incidences of overall complications with the ISB, as observed in our study [33,34,37].

In contrast, the incidence of phrenic nerve paralysis with STB was substantially lower in our meta-analysis. Total hemidiaphragmatic paralysis occurred in 10 of 178 (5.6%) patients who underwent STB. Robles et al. reported higher diaphragmatic effects among 30 patients who had STB, of whom 33.3% had total hemidiaphragmatic paralysis [38].

Both the STB and ISB demonstrated a low overall rate of complications. However, the incidence of Horner syndrome was found to be significantly higher in the ISB compared to the STB. Similar findings have been reported in the literature [34,39,40]. This outcome is expected with the ISB because of the potential spread of local anesthetic to the nearby stellate ganglion, which can lead to ipsilateral sympathetic cervical chain paralysis [41]. No major neurological complications, local anesthetic systemic toxicity, or other relevant complications were observed with either technique.

In our study, both strategies had similar analgesia time, opioid consumption, and overall patient satisfaction with analgesia. However, STB yielded significantly better pain scores at 24 hours than ISB. Importantly, this finding should be interpreted in light of clinical relevance rather than statistical significance. The mean difference of 0.75 mm between groups does not meet the minimum clinically important difference threshold for this particular outcome, pre-established at 11 mm. Therefore, while the STB may have a statistical advantage in this outcome, caution should be exercised when interpreting this finding.

Study Limitations

The relatively small sample size may have resulted in limited statistical power to detect minor differences between groups. However, those minor differences, even if they exist, are likely to be clinically irrelevant, given the confidence interval of our results, as discussed. Moreover, our study represents the largest sample size in RCTs comparing these techniques. Therefore, it is unlikely that new studies would significantly alter the clinical implications of our meta-analysis.

Also, the similarity in some of the outcomes between the included RCTs may question the need for this meta-analysis. However, pooling the results of the individual RCTs allowed us to increase the sample size and statistical power of the sample, resulting in narrower confidence intervals and more precise estimates of treatment effects. Based on our findings, further studies investigating the comparative efficacy and safety of STB versus ISB are likely unnecessary.

Additionally, some outcomes had high heterogeneity (I2>25%). This increased heterogeneity may be due to differences in block techniques among studies, including, but not limited to, the anesthetic volume, concentration, and agent of choice for the block (Table 1). Operator experience may also have been variable between different studies. Finally, the publication bias analysis using the Doi plot and LFK index suggests some degree of asymmetry in the data, hinting at the possibility of small study effects. However, this asymmetry may not solely be due to publication bias, as various factors, such as differences in study quality, baseline risks in small and large studies, or even random chance, could contribute to this asymmetry. Therefore, it would be overly simplistic to conclude that publication bias is the primary explanation for the observed asymmetry in this specific case.

Our meta-analysis has significant implications for clinical practice. The superior safety profile of STB, combined with similar analgesic efficacy to ISB, makes it a compelling option for patients undergoing shoulder arthroscopy, particularly those with coexisting respiratory conditions such as severe chronic obstructive pulmonary disease, obstructive sleep apnea, or morbid obesity, among others [42,43].

## Conclusions

In this meta-analysis of RCTs including patients undergoing shoulder arthroscopy, STB was found to have equivalent analgesic efficacy compared with ISB, albeit with a superior safety profile in terms of respiratory outcomes.

Therefore, STB should be considered a preferred alternative to ISB, particularly for patients at increased risk of respiratory insufficiency.
